# Her2-Positive Cancers and Antibody-Based Treatment: State of the Art and Future Developments

**DOI:** 10.3390/cancers13225771

**Published:** 2021-11-18

**Authors:** Serafin Morales, Ariadna Gasol, Douglas Rene Sanchez

**Affiliations:** 1 Medical Oncology Department, Hospital Universitari Arnau de Vilanova de Lleida, 25198 Lleida, Spain; ariadna.gc.86@gmail.com; 2 Pathology Department, Hospital Universitari Arnau de Vilanova de Lleida, 25198 Lleida, Spain; douglas.pathology@gmail.com

**Keywords:** HER2 positive breast cancer, targeted therapies, treatment algorithm

## Abstract

**Simple Summary:**

HER2 positive breast cancer has been increasingly researched and its management improved. In all settings, it has been possible to improve both survival and quality of life with less aggressive and more effective treatments. The appearance of new drugs has made it possible to control the disease in advanced and highly compromised stages, achieving very high expectations of efficacy.

**Abstract:**

HER2 positive breast cancer represent about 20% of all breast cancer subtypes and it was considered the subtype with the worst prognosis until the discovery of therapies directed against the HER2 protein. The determination of the status of the HER2 must be very precise and well managed to identify this subtype, and there are very specific and updated guides that allow its characterization to be adjusted. Treatment in local disease has been considerably improved with less aggressive and highly effective approaches and very high cure rates. In metastatic disease, average median survival rates of 5 years have been achieved. New highly active molecules have also been discovered that allow disease control in very complicated situations. This article reviews all these options that can be used for the management of this disease.

## 1. Diagnoses

The human epidermal growth factor receptor 2 HER2/neu (c-erbB-2) gene is located on the long arm of chromosome 17 and encodes the HER2 protein, a transmembrane receptor with tyrosine kinase activity [1]. HER2 belongs to the epidermal growth factor receptor (EGFR) family, also called the HER family. This family is composed of four members (HER1 to HER4), and plays, under physiological conditions, a role in intercellular communication, and between cell and stroma [2,3]. However, HER receptors exhibit abnormal signaling activity in a wide range of tumors. Within this family, HER2 hyperactivity is particularly oncogenic [4].

The lines of evidence that prompted consideration of HER2 as a therapeutic target were the following: transfection of the HER2 gene induces the malignant phenotype; HER2 is overexpressed in 17–20% of human breast cancers; the main cause of HER2 overexpression is gene amplification; HER2 overexpression or gene amplification carries a poor prognosis in patients with breast cancer [5]; and finally, at the end of the 1990s it was shown that monoclonal antibodies directed against HER2 were capable of exerting an antitumor effect. One of these antibodies, the murine 4D5 antibody, was particularly active in HER2 overexpressing cell lines or tumors. Humanization of 4D5 resulted in the anti-HER2 antibody trastuzumab (Herceptin, Roche, Bassel) [6].

The American Society of Clinical Oncology (ASCO) and the American College of Pathologists (CAP) state that the HER2 test should be performed in all cases of invasive breast carcinomas [7].

**Figure 1:**
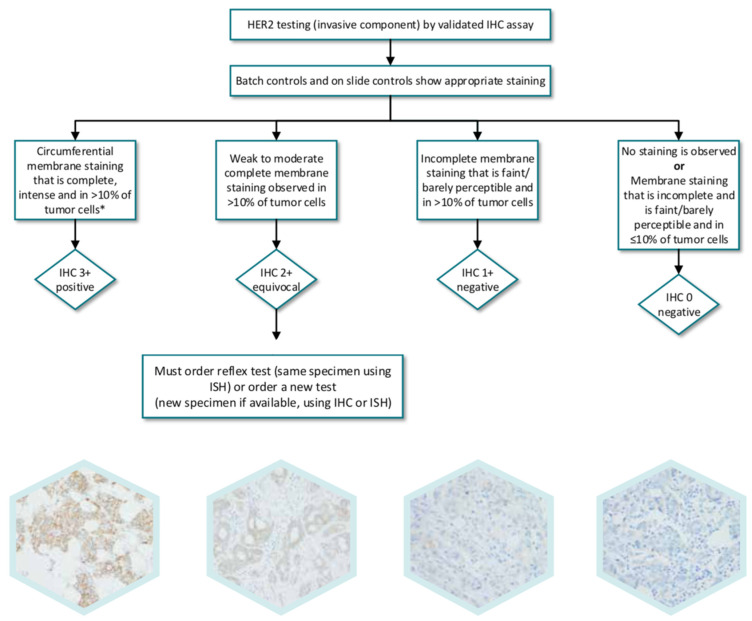
Algorithm for evaluation of human epider mal growth factor receptor (HER2) protein expression by immunohistochemistry (IHC) assay of the invasive component of a breast cancer specimen.

The best method to determine HER2 status and diagnostic algorithms are still controversial in 2021 and one of the most important immunohistochemical studies to be performed in breast cancer is the HER2/neu test [8]. Interpretation of results should be done by a pathologist. The evaluation should be done exclusively on the infiltrating component and only the membrane staining will be evaluated (Figure 1).

**Figure 2:**
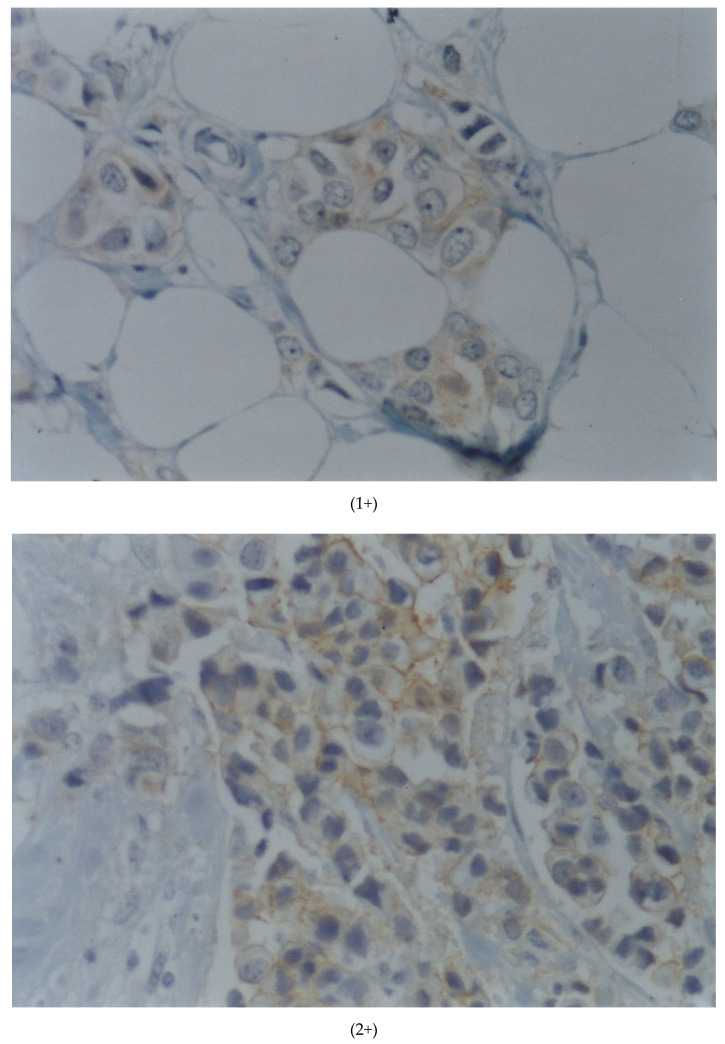

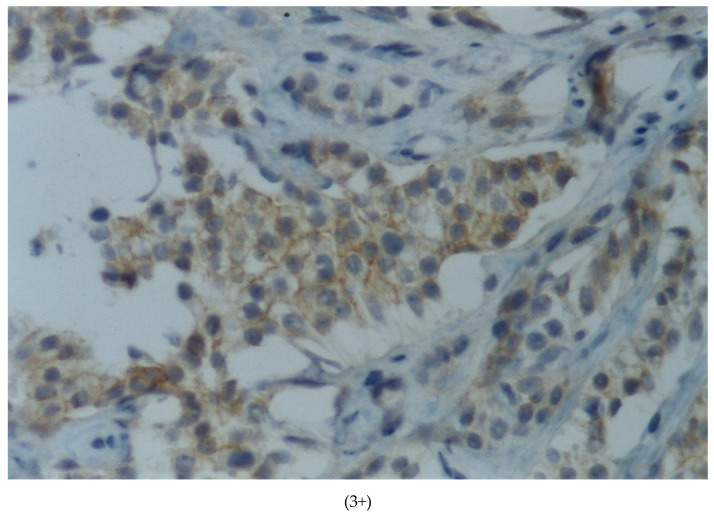
Immunohistochemical images of her2. scale bar or magnification: 25× in (1+) and 20× in (2+) and (3+).

A negative result (0/1+) will be considered in the absence of membrane staining or staining in less than 10% of cells (0), or if membrane staining is weak and incomplete in more than 10% of cells (1+). A positive result (3+) will be considered when there is complete and intense membrane staining in more than 30% of the cells [7,8]. An indeterminate result (2+) will be considered when there is complete membrane staining, weak or moderate, in more than 10% of the cells or complete and intense staining in 10–30% of the cells (Figure 2). It should be emphasized that if most of the cells show incomplete membrane staining, but cells with complete staining are also observed and represent more than 10%, but less than 30%, the result is 2+. Even though it is not expressly included in international guidelines, those cases are of difficult interpretation due to mild fixation artifacts or slight over-unmasking with discrete staining of normal mammary epithelium, and also those in which that there is intense but not complete membrane staining [9].

There are currently a number of image analyzers that can assist in the interpretation of these immunohistochemical tests. These methods have been developed to achieve a more precise interpretation, other than an operator-dependent one. Multiple studies have been carried out comparing both techniques, in which it has been found that automated image analyzers can be a good alternative for diagnosis [10].

In a study carried out in Cairo, the results of image analysis were compared with those of FISH in cases in which the HER2 was indeterminate (2+). In this study, 15 cases previously reported as indeterminate HER2 (2+) were taken and evaluated using an image analyzer (Roche iScan Coreo), three of them were positive (3+) in the image analyzer and of these two were also positive on FISH. Those that remained 2+ after automated analysis were negative on FISH. It was concluded that image analysis is highly sensitive and specific, similar to FISH for detecting HER2 overexpression, so it can be used in cases of immunohistochemistry evaluated manually and reported as 2+, before resorting to FISH tests. 

**Figure 3:**
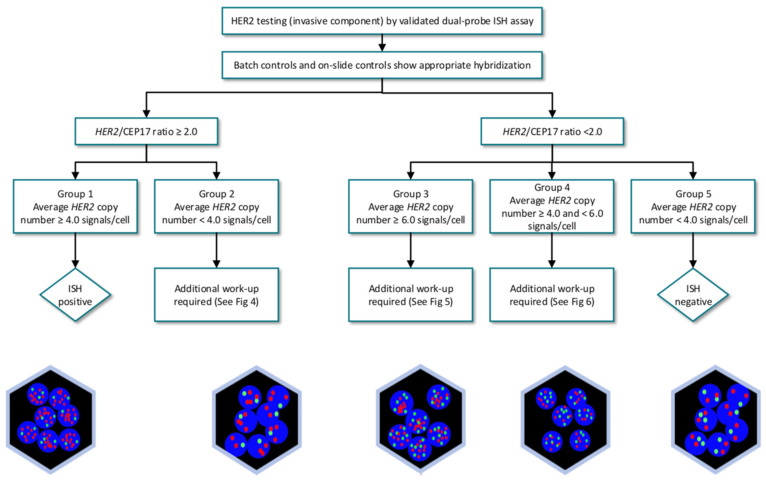
Algorithm for evaluation of human epider mal growth factor receptor (HER2) gene amplification by in situ hybridization (ISH) assay of the invasive component of a breast cancer specimen use a dual-signal (HER2 gene) assay (dual-probe ISH).

For the interpretation of the FISH, the following cut-off points are used according to the updated recommendations of ASCO/CAP of 2013 on the determination of HER-2 [11]: (Figure 3).

**Normal or non-amplified levels:** HER2/chromosome 17 ratio <2.0 and average HER2 copies <4.0 signals per cell.

**Misleading levels:** HER2/Chromosome 17 ratio <2.0 and average HER2 copies> or equal to 4.0 but <6.0 signals per cell.

**Amplified levels:** HER2/Chromosome 17 ratio> or equal to 2.0 (with the average number of HER2 copies> or equal to 4.0 or <4.0) or average HER2 copies> or equal to 6.0 signals/cell (regardless of the HER2/Chromosome ratio 17).

Other studies have shown that the results obtained using an image analyzer have better agreement with FISH than manual interpretation [12,13,14,15,16].

Determining the magnitude of HER2/neu positivity is very important for prognosis and treatment, although its interpretation is usually observer-dependent [17]. For this reason, an attempt is made to find other forms of interpretation, such as image analyzers, but it is important to carry out the validation [18].

There are even image analyzers in which the sensitivity of detection of the intensity of the immunohistochemical staining can be modified, which makes it adaptable to the personal characteristics of the technique of each laboratory [19]. If these new assistive techniques are to be used for the interpretation of immunohistochemistry, the most important thing is definitely to validate their use in order to have consistent results.

## 2. Early Breast Cancer

Neoadjuvant setting provides the opportunity to test in vivo the response to the treatment and could help to identify potential predictive factors and biomarkers. Neoadjuvant chemotherapy in HER2 positive breast cancer has an impact on overall survival derived especially in tumors with complete pathological response (pCR) [20]. Neoadjuvant scenario constitutes a unique opportunity to test new drugs and alternative treatments when primary systemic therapy is not sufficiently effective and could provide tools to complement treatment in those without response (examples: adjuvant trastuzumab emtansine in HER2 positive residual disease [21] or capecitabine in residual triple negative [22]). Achievement of pCR is an acceptable surrogate endpoint of clinical benefit; thus, the FDA had accelerated approval of some new drugs with a clinical advantage achieving pCR.

HER2 disease is quite chemosensitive, in fact, pCR rates reported in The CTNeoBC pooled analysis [20] showed that pCR rates in HER2 positive disease and estrogen receptor positive (ER) with neoadjuvant chemotherapy with or without trastuzumab was 30.9% versus 18.3%, pCR in HER2 positive and ER negative with or without trastuzumab was 50.3% versus 30.2%. These data suggest the need of some antiHER2 therapy added to neoadjuvant chemotherapy in order to increase pCR rates.

The adjuvant setting is relegated for smaller tumors and/or those tumors where the initial tumor size was less than 1 cm and after surgery it becomes higher. Adjuvant options will be reported later in this article.

Since trastuzumab has changed the natural history of HER2 positive breast cancer, there could not be any HER2 positive patient without this treatment in the multidisciplinary approach because of better outcome benefits already demonstrated. In general, international guidelines suggest that patients with HER2 positive disease who had a tumor T2 (more than 2 cm), or nodal involvement should receive neoadjuvant chemotherapy plus antiHER2 therapy. After all the clinical benefits of neoadjuvant treatment were published, all HER2 positive tumor subsidiary for chemotherapy should be administered in the neoadjuvant setting. According to this premise, a review of the main data published will be summarized and after that, a summing up table will be provided.

The NOAH trial randomized 235 HER2 positive breast cancer patients to receive neoadjuvant chemotherapy with or without trastuzumab, with an increase from 22% with chemotherapy alone to 43% pCR with addition of trastuzumab (*p* = 0.0007) [23]. The 5-year event-free survival was 58% (95% CI 48–66) in patients in the trastuzumab group and 43% (95% CI 34–52) in those in the chemotherapy group; the unadjusted HR for event-free survival between the two randomized HER2-positive treatment groups was 0.64 (95% CI 0.44–0.93; two-sided log-rank *p* = 0.016). Event-free survival was strongly associated with pathological complete remission in patients given trastuzumab. Of the 68 patients with a pCR (45 with trastuzumab and 23 with chemotherapy alone), the HT for event-free survival between those with and without trastuzumab was 0.29 (95% CI 0.11–0.78) [24].

The addition of new antiHER2 drugs had provided better pCR rates, however not all of them are approved by agencies. In the neoadjuvant NeoALTTO trial dual HER2 blockade with lapatinib plus trastuzumab combined with weekly paclitaxel significantly increased the pCR rate compared with either anti-HER2 agent alone plus paclitaxel (pCR of 51.3%, 95% CI 43.1–59.5 in the group with lapatinib and trastuzumab; pCR of 29.5%. 95% CI 22.4–37.5 in the trastuzumab alone group, difference 21.1%, *p* = 0.0001) [25].

A better approach has been seen with the introduction of Pertuzumab. In the NeoSphere trial patients given pertuzumab and trastuzumab plus docetaxel had a significantly improved pCR rate (45.8%, 95 CI 36.1–55.7) compared with those given trastuzumab plus docetaxel (29%, 95% CI 20.6–38.5; *p* = 0.0141) [26]. Updated survival analysis showed that patients who achieved total pathologically complete response (all groups combined) had longer progression-free survival compared with patients who did not (85% [95% CI 76–91] in patients who achieved total pathological response vs. 76% [95% CI 71–81] in patients who did not achieve total pathological response; hazard ratio 0·54 [95% CI 0.29–1.00]) [27]. Combinations of pertuzumab and trastuzumab and random chemotherapy agents, also demonstrated an increase on pCR rate with pertuzumab.

The TRYPHAENA trial was a multicenter, open-label phase II study were patients with operable, locally advanced, or inflammatory breast cancer randomized 1:1:1 to receive six neoadjuvant cycles q3w (Arm A: 5-fluorouracil, epirubicin, cyclophosphamide [FEC] + trastuzumab + Pertuzumab × 3 → Docetaxel + trastuzumab + pertuzumab × 3; Arm B: FEC × 3 → Docetaxel + trastuzumab + Pertuzumab × 3; Arm C: Docetaxel + Carboplatin + Trastuzumab + Pertuzumab × 6). The primary endpoint was cardiac safety with no differences in symptomatic left ventricle systolic dysfunction. pCR was reported for 61.6% (Arm A), 57.3% (Arm B), and 66.2% (Arm C) of patients [28]. NeoSphere’s results, in combination with the results from the neoadjuvant TRYPHAENA study, led the US Food and Drug Administration in 2013, and the European Medicines Agency in 2015, to grant pertuzumab accelerated approval in the neoadjuvant setting, making pertuzumab the first drug to be approved using pathologically complete response as an endpoint.

Other strategies escalating or de-escalating chemotherapy or Pertuzumab, have been studied in the neoadjuvant setting, with similar pCR rates than those reported with Pertuzumab. Some of them, suggest a selection of patients according to other tools in order to decide the need of more or less neoadjuvant therapy.

The ADAPT trial, which selected baseline features of HER2-positive HR negative patients that were randomized to 12 weeks of Trastuzumab + Pertuzumab +/− weekly paclitaxel at 80 mg/m^2^ and had an early response (proliferation decrease ≥30% of ki67 or <500 invasive tumor cells in the 3-week biopsy). Responder patients had a pCR rate in the Trastuzumab + Pertuzumab + paclitaxel arm of 90.5%, compared with 36.6% in the Trastuzumab + Pertuzumab arm. These results suggest that some chemotherapy is need in responder patients, however, if they respond, a less toxic chemotherapy strategy could be proposed [29] with only paclitaxel. New data updated in ASCO 2021, reported that the achievement of a pathologically complete response (vs. not) after the 12-week study treatment was strongly associated with improved invasive disease-free survival at 5 years, irrespective of study arm: 98% vs. 82% (HR = 0.14; *p* = 0.11) [30].

Hormone receptor-positive Her2-positive tumors usually have less response to standard neoadjuvant chemotherapy, so ADAPT HER2+/HR+ aimed to identify responders to dual targeted therapy (endocrine and antiHER2). 380 patients were randomized to receive 12 weeks of TDM1 (trastuzumab − emtansine) +/− endocrine therapy (Arms A/B) or trastuzumab + endocrine therapy (Arm C). Overall pCR rate was 30.8%, Arm A: 40.5%, B: 45.8%, C: 6.7%, with significant difference between arms with TDM1 vs. not (*p* < 0.001), but not between TDM1 arms. Exploratory analysis suggests benefit of adding endocrine therapy to TDM1 in premenopausal (pCR: 28.6% for TDM1 single agent vs. 47.6% with endocrine therapy) but not in postmenopausal patients (pCR: 64.3% vs. 50%). Hence it needs further investigation; TDM1 could become another option for HR+ HER2+ patients, but not approved yet by FDA [31].

Trastuzumab-Emtansine has been tested in the neoadjuvant setting compared to trastuzumab and pertuzumab + chemotherapy in a Kristine clinical trial with a 44.4% of pCR in the group with TDM1 + pertuzumab versus 55.8% pCR in patients treated with carboplatin + docetaxel + trastuzumab + pertuzumab, suggesting that TDM1 did not make any difference in pCR rate in the whole population, although a better toxicity profile was reported. TDM1 neither showed benefit in pCR rates in the HR positive patients, with a pCR of 35.1% for TDM1 + pertuzumab versus 43.8% pCR with chemotherapy + trastuzumab + pertuzumab (CI 95% −20.5–3.2%) [32]. In an exploratory multivariate logistic regression analysis to control for clinicopathologic factors, treatment and HR status were associated with archiving a pCR. T-DM1+P treatment (OR = 0.62; 95% CI 0.42, 0.93) and positive HR status (OR = 0.43; 95% CI 0.28, 0.65) led to lower odds of achieving a pCR. TDM1 in the whole HER2 population which is not a good approach for increasing pCR rate, and any subgroup showed clinically meaningful benefits.

A more daring approach is chemotherapy-free regimens, with only double anti-HER2 therapy, but this approach needs a selection (in advance) of patients who could achieve a pCR without chemotherapy. The PHERGain trial evaluated if early metabolic responses to chemotherapy-free regimens with only neoadjuvant trastuzumab and pertuzumab measured with changes in 18F-fluorodeoxyglucose (18F-FDG)-PET uptake could select those who do not need chemotherapy. Patients were randomized (1:4) to receive either docetaxel, carboplatin, trastuzumab and pertuzumab (group A); or trastuzumab and pertuzumab (group B); HR positive patients also received endocrine therapy. 18F-FDG-PET were performed before randomization and after two cycles. Patients in the chemotherapy group (A) received all 6 cycles of pre-planned chemotherapy regardless of 18F-FDG responses; however, for patients in group B (chemotherapy-free), if they responded by 18F-FDG-PET, they continued with the same regimen and if they did not respond, they were switched to six cycles of chemotherapy similar to group A. At 2–6 weeks after completion of the study, surgery was performed and adjuvant treatment was administered according to previous neoadjuvant treatment, pathological response, hormone receptor status and clinical stage at diagnosis; 227 of 285 (80%) of patients in group B (without chemotherapy) had a 18F-FDG-PET response, of whom 86 of 227 (37.9%) had a pathological complete response versus 25.9% of pCR in non-responders (*p* = 0.068). In group A with chemotherapy, the global pCR rate was 57.7%; 65.6 in 18F-FDG-PET responders versus 10% in non-responders (*p* = 0.013) [33]. Hence, 18F-FDG-PET response by variation of uptake could select patients who will have greater pCR rates, however, chemotherapy is still needed in combination with antiHER2 therapies.

After PHERGain trial pCR rates, there is still a need to better select those patients who could avoid chemotherapy, although 37.9% is not a good standard in this phenotype of patients. The Phergain 2 trial is ongoing with a rationale that in group B/responders, patients with HER2 expression 3+ by immunohistochemistry (IHC) (*n* = 184) had a better pCR rate than patients with HER2 IHC 2+ and HER2 gene amplification by an in situ hybridization assay (ISH) (*n* = 43); pCR in HER2 3+ IHC 40.3% vs. 25.6% if HER2 2+ IHC (*p* = 0.003). Therefore, PHERGain-2 trial selected patients with HER2 3+ IHC and tumors from 5 mm to 25 mm measured by MRI, without initial node involvement, to receive neoadjuvant treatment with eight cycles of fixed-dose subcutaneous trastuzumab and pertuzumab combination and endocrine therapy according to HR status [34].

Many trials have been initiated to try to establish which chemotherapy drug(s) are the most effective to use in combination with trastuzumab and pertuzumab. Better pCR rates were reported in the GEPARSEPTO trial which compared the use of nab-paclitaxel versus solvent-based paclitaxel. In the HER2 positive cohort 123 of 199 patients (62%) achieved a pathological complete response with nab-paclitaxel compared with 106 of 197 (54%) of solvent-based paclitaxel (*p* = 0.13; p interaction = 0.31). Patients with the biological subtype of HER2-positive/HR-positive disease were 56% versus 50% (*p* = 0.30); and with HER2-positive/HR-negative were 75% versus 67%, respectively (*p* = 0.49; p interaction = 0.20) [35].

In order to find the need or anthracyclines or not in combination with dual HER2 blockade in the neoadjuvant setting, and whether its addition would improve pCR compared with a carboplatin-taxane regimen, this was evaluated in the phase 3 TRAIN-2 [36]. Patients were randomly allocated (1:1) to receive 5-fluoracil (500 mg/m^2^), epirubicin (90 mg/m^2^), and cyclophosphamide (500 mg/m^2^) every 3 weeks for three cycles followed by paclitaxel 80 mg/m^2^ on days 1 and 8) and carboplatin (AUC 6 mg/m^2^) every 3 weeks for six cycles, or to receive nine cycles of paclitaxel and carboplatin at the same dose and schedule. Patients in both study groups received trastuzumab and pertuzumab concurrently with all chemotherapy cycles. A pCR was recorded in 67% (95% CI 60–73) of the anthracycline group and in 68% (95% CI 61–74) of the non-anthracycline group (*p* = 0.95), with no differences and symptomatic left ventricular systolic dysfunction was rare in both groups. In conclusion, this study suggests that the use of anthracycline does not add cardiotoxicity but has similar pCR rate of carboplatin-taxane regimen, so if a patient does not receive anthracycline should receive instead a carboplatin-taxane regimen.

Chemotherapy and antiHER2 treatment in the adjuvant setting will be relegated for those patients who were not diagnosed with a more than 1 cm disease, because the clinician would mainly administer chemotherapy to all patients at diagnosis because of clinical features, and this treatment should be planned in the neoadjuvant setting. However, antiHER2 treatment should be continued, and right now, the standard of care remains the HERA strategy, to complete 1 year of trastuzumab or a total of 18 cycles. At 11 years of follow-up, 1 year of trastuzumab significantly reduced the risk of a disease-free survival event (HR 0.76, 95% CI 0.68–0.86) and death (0.74, 0.64–0.86) compared with observation; and 2 years of adjuvant trastuzumab did not improve disease free-survival outcomes compared with 1 year (HR 1.02, 95% CI 0.89–1.17) [37].

Other strategies of de-escalation for the duration of adjuvant trastuzumab failed to demonstrate non-inferiority [38]. However, in the PERSEPHONE phase 3 trial, 4 year disease-free survival was 89.4% (95% CI 87.9–90.7) in the 6-month group and 89.8% (95% CI 88.3–91.1%) in the 12-month group (HR 1.07 [90% CI 0.93–1.24], non-inferiority *p* = 0.011), showing non-inferiority of the 6-month treatment. Persephone also reported less cardiotoxicity, which could support the idea of only 6-months of trastuzumab treatment in those patients in risk of cardiac toxicity [39].

Only 12 weeks of paclitaxel plus trastuzumab in small tumors is currently used, either in neoadjuvant or adjuvant settings according to phase 2 trial of Tolaney et al. [40]. Patients with HER2-positive breast cancer tumors 3 cm or smaller and negative nodes received paclitaxel (80 mg/m^2^) with trastuzumab for 12 weeks, followed by trastuzumab for 9 months. The 7-year disease-free survival was 93% (95% CI, 90.4 to 96.2) with four (1%) distant recurrences, 7-year overall survival was 95% (95% CI, 92.4 to 97.7), and 7-year recurrence free interval was 97.5% (95% CI, 95.9 to 99.1). These data strongly support a routine clinical practice and that in those tumors, only one antiHER2 to drug could be safely used.

Double antiHER2 blockage with trastuzumab and pertuzumab had been studied in the APHINITY trial [41]; 4805 patients with node-positive or high-risk node-negative HER2-positive, operable breast cancer were randomly assigned to receive either pertuzumab or placebo added to standard adjuvant chemotherapy plus 1 year of treatment with trastuzumab; 63% had node-positive disease and 36% were hormone receptor negative. The 3-year rates of invasive-disease-free survival were 94.1% in the Pertuzumab group and 93.2% in the placebo group.

In the cohort of patients with node-positive disease, the 3-year rate of invasive-disease-free survival was 92.0% in the pertuzumab group, as compared with 90.2% in the placebo group (HR 0.77; 95% CI, 0.62 to 0.96; *p* = 0.02). In the cohort of patients with hormone-receptor-negative tumors, the 3-year rate of invasive-disease-free survival was 92.8% in the pertuzumab group and 91.2% in the placebo group (HR 0.76; 95% CI 0.56 to 1.04; *p* = 0.08). With these results, addition of pertuzumab to adjuvant chemotherapy plus trastuzumab, only derives a small benefit, mainly in those tumors with poorer prognosis such as node-positive or HR negative.

These results were updated after 6 years of follow up and 6-year overall survival were 95% versus 94%, respectively, without a statistical significance (*p* = 0.17, HR 0.85). Invasive-disease-free survival (IDFS) was of 91% versus 88% for pertuzumab and placebo groups, respectively. The node-positive cohort continues to derive clear IDFS benefit from pertuzumab (HR 0.72 [95% CI, 0.59 to 0.87]), 6-year IDFS being 88% and 83%, respectively. Benefit was not seen in the node-negative cohort. In a subset analysis, IDFS benefit from pertuzumab showed a HR of 0.73 (95% CI, 0.59 to 0.92) for HR-positive disease and a hazard ratio of 0.83 (95% CI, 0.63 to 1.10) for HR-negative disease [42].

With these data, it seems that the longer-term magnitude of benefit of adding pertuzumab to standard adjuvant therapy may not depend upon hormone receptor status of the primary tumor; the best benefit could be added in the node-positive cohort. Taking into account that in clinical practice almost all HER2-positive tumors will receive neoadjuvant treatment, the APHINITY approach will be reserved for those tumors that were small at diagnosis and not candidates for neoadjuvant treatment, and after surgery a node involvement is found.

Adjuvant treatment after neoadjuvant treatment should be planned according to benefit obtained after neoadjuvant approach; meaning whether a pCR is achieved or not. Because of higher IDFS rates in the non-pCR group after neoadjuvant treatment, new anti-HER2 agents have been tested in this setting in order to improve invasive-disease free survival. T-DM1 was tested in the phase 3 Katherine trial, involving patients with HER2-positive early breast cancer who were found to have residual invasive disease in the breast or axilla at surgery after receiving neoadjuvant therapy containing a taxane (with or without anthracycline) and trastuzumab. 1486 patients were randomly assigned to receive adjuvant T-DM1 or trastuzumab for 14 cycles. IDFS was significantly higher in the TDM1 group (3-year IDFS 88.3%) than in the trastuzumab group (3-year IDFS 77%) (HR 0.50; 95% CI, 0.39 to 0.64, *p* < 0.001) [21]. Adjuvant TDM1 could improve survival in those patients without a pCR after neoadjuvant treatment.

The use of Neratinib, an irreversible pan-HER tyrosine kinase inhibitor, given for 1 year after completion of trastuzumab was investigated in the phase III ExteNET trial, and was shown to significantly improve invasive disease-free survival compared with placebo at the planned primary analysis time point of 2 years (HR 0.66, 95% CI 0.49–0.90, *p* = 0.008) [43]. The efficacy of neratinib was confirmed at the 5-year analysis (HR 0.73; 95% CI, 0.57–0.92; *p* = 0.008), and was more marked in patients who initiated treatment within 1 year of completing prior trastuzumab and among patients with hormone receptor positive.

The greater efficacy of Neratinib in those who receive concurrent endocrine therapy may be attributed to the effective inhibition of cross-talk between HER2 and estrogen receptors, as HER2-positive/HR-positive tumors are at continuous risk of late recurrences. In the HER2-positive/HR-positive population, IDFS rates at 2 years were 95.3% (95% CI, 93.1–96.75%) with neratinib and 90.8% (95% CI, 88.2–92.9%) with placebo, corresponding to an absolute benefit of 4.5% with Neratinib (HR 0.49; 95% CI, 0.30–0.78). In the 5-year analysis, IDFS rates were 90.8% (95% CI, 88.1–93.0) in the Neratinib group and 85.7% (95% CI, 82.6–88.3) in the placebo group, corresponding to a durable absolute benefit or 5.1% (HR 0.58; 95% CI 0.41–0.82).

It is of note that in patients with no pCR after neoadjuvant treatment, 5-year IDFS rates were 85% (95% CI 77–90.4) in the Neratinib group and 77.6% (95% CI 69.8–83.6) in the placebo group, corresponding to an absolute benefit of 7.4% (HR 0.60; 95% CI 0.33–1.07) [44]. The use of Neratinib for 1 year as a second adjuvant antiHER2 therapy will be in those tumors that are HER2-positive/HR-positive with high-risk or those with residual disease after neoadjuvant treatment. We proposed a treatment algorithm with all the information reported above in the Table 1.

## 3. Advanced Breast Cancer

Since Slamon et al. [45] published in 2001 the results of the combination of chemotherapy with trastuzumab, the treatment paradigm for HER2-positive breast cancer has changed. HER2 positive metastatic breast cancer has achieved a significant prolonged survival, and the development of many anti-HER2 agents has led to unprecedented survival outcomes.

### 3.1. Treatment in First Line

The combination of taxane with trastuzumab and pertuzumab (CLEOPATRA TRIAL [46]: placebo plus trastuzumab plus docetaxel (control group) or pertuzumab plus trastuzumab plus docetaxel (pertuzumab group)) has shown impressive survival rates in patients with metastatic HER2-positive disease in the first line of treatment. After a median follow-up of 99.9 months [47], a median overall survival of 57.1 months in the pertuzumab group vs. 40.8 in the control group (without pertuzumab) was observed. This supposes an improvement of 16.3 months and a risk for death decreased by 31% (HR for overall survival: 0.68 95% CI, 0.56–0.84; *p* < 0.001).

Pertuzumab was well tolerated, and the toxicity was very low, with diarrhea, skin rash, headache, and muscle spasm being the most relevant. Nor were differences observed in cardiotoxicity, which was also very low [48]. With these data, the trastuzumab, pertuzumab, and taxane regimen were established as the standard first-line treatment in patients with HER2-positive MBC [49].

Although the analysis of overall survival in predefined subgroups indicated a consistent survival benefit, it is important to analyze the results in these subgroups. The majority of the patients were confirmed HER2 positive (91%), half were estrogen-receptor positive, and 77% had visceral disease. It is interesting that the 50% not received previous adjuvant or neoadjuvant therapy [46].

In the subgroup analysis, it was observed that the 88 patients who received prior treatment with neoadjuvant or adjuvant trastuzumab had an overall survival benefit with a hazard ratio of 0.68 (95% CI 0.30 to 1.55).

The early relapse subgroup was not represented in the CLEOPATRA trial, being a subgroup with a particularly poor prognosis [50]. However, the combination treatment with trastuzumab plus pertuzumab was approved in the first line regardless of the relapse time. In the EMILIA trial [51] (T-DM1 versus lapatinib plus capecitabine), this subgroup was included in a small proportion (15%), finding a benefit in favor of treatment with T-DM1, therefore the regulatory agencies approved the treatment with T-DM1 in this subgroup [49].

### 3.2. Improvement in This Area: Results of pi3ka Mutations

PIK3CA mutation is a signaling oncogenic that activates the (PI3K)/AKT/mTOR pathway and acts as an oncogenic driver and regulates cell growth, proliferation, survival, differentiation, angiogenesis and many other cell functions.

In the analysis of biomarkers with effect on survival benefit, PIK3CA was the only biomarker that showed prognostic effect, with longer median disease free survival (DFS) for patients whose tumors expressed wild-type versus mutated PIK3CA in both the control (13.8 vs. 8.6 months) and pertuzumab groups (21.8 vs. 12.5 months) [52]. Although patients with PIK3CA mutations also benefit from the combination with pertuzumab, the DFS is only 12 months. PIK3CA mutation identifies patients with high unmet needs, despite deriving DFS benefit from treatment with pertuzumab plus trastuzumab plus docetaxel. Thus, new trials combining HER2-targeted therapy and PIK3 inhibitors are currently underway.

There are some trials to investigate the role of PIK3CA inhibitors to improve the results in patients with PIK3CA mutations. One interesting trial is the Solti-1507, it is a phase Ib study of ipatasertib and anti-HER2 therapy in her2-positive advanced breast cancer with PIK3CA mutation (ipather) [53].

### 3.3. Improvement in This Area: Combination with Endocrinotherapy

Another approach is the use of antiHER2 therapy with endocrine-therapy in patients with hormonal receptor expression. In the CLEOPATRA trial, the benefit in overall survival for positive estrogen receptor was worse than negative estrogen receptors (HR 0.73 vs. HR: 0.57), so its addition to endocrine therapy could be an attractive approach [47]. Also, the CLEOPATRA trial does not allow maintenance endocrine therapy. Thus, the value of endocrine therapy in this setting is unknown.

In the PERTAIN trial [54], it was hypothesized that pertuzumab, trastuzumab, and an endocrine-therapy (aromatase inhibitor) may offer additional benefits compared with trastuzumab plus an aromatase inhibitor for HER2–positive and hormone receptor–positive MBC or locally advanced breast cancer (LABC) in first line. Induction intravenous docetaxel every 3 weeks or paclitaxel every week could be administered for 18 to 24 weeks at the investigator’s discretion.

The median DFS was 18.89 months (95% CI, 14.09 to 27.66 months) in the pertuzumab plus trastuzumab arm and 15.80 months (95% CI, 11.04 to 18.56 months) in the trastuzumab arm (stratified hazard ratio, 0.65; 95% CI, 0.48 to 0.89; *p* = 0.0070). In patients who did not receive induction chemotherapy, the pertuzumab plus trastuzumab showed a longer DFS (21.72 months) than trastuzumab arm (12.45 months; unstratified HR: 0.55; 95% CI, 0.34 to 0.88). Whereas patients who received induction chemotherapy the DFS was similar (16.8 months vs. 16.85; unstratified HR: 0.75; 95% CI, 0.50 to 1.13). Finally authors concluded that pertuzumab plus trastuzumab and an aromatase inhibitor is effective for the treatment of these patients.

### 3.4. Improvement in This Area: T-DM1 in First Line MBC

Treatment with T-DM1 in the first line is not indicated except for the early relapse subgroup, which has been previously commented upon, based on the results in favor of T-DM1 in the global series, but the benefit of T-DM1 in this subgroup is unknown.

The role of first-line T-DM1 was considered in the MARIANNE phase III randomized controlled trial, [55] which compared T-DM1, alone or with the combination of pertuzumab, trastuzumab, and taxane. This study showed a non-inferiority of TDM compared to the combination of pertuzumab, trastuzumab and taxane. Other series with retrospective studies [56] also showed poorer survival with T-DM1 compared to the combination of trastuzumab plus pertuzumab. Therefore, the combination of trastuzumab + pertuzumab + docetaxel remains the best first-line treatment option for metastatic breast cancer.

### 3.5. Improvement in This Area: Other First-Line Approaches

New active drugs and special scenarios has been studied and compared to standard treatment with the combination to trastuzumab plus pertuzumab. Trastuzumab deruxtecan and pyrotinib are very promising drugs that are being investigated their activity in first line treatment. New treatment with immune checkpoint inhibitors such as atezolizumab is also being studied in this situation. Finally, special situations such as positive receptors or brain metastasis are being investigated with specific therapy.

Other conditions that are being investigated are maintenance after a first line of standard treatment, such as the aforementioned maintenance with hormonal treatment (PERTAIN trial) or with AKT inhibitors (IPATHER). Moreover, the role of cyclin inhibitors (PALBOCICLIB) in this setting is currently being studied. Table 2 summarizes trials that are ongoing in these conditions.

### 3.6. Treatment in Second Line

The data with T-DM1 are very consistent for patients who progress to a first line. T-DM1 is an antibody-drug conjugate that is composed of an antibody targeting HER2 conjugated via a non-cleavable linker to DM1, an emtansine analogue that inhibits microtubules. We have very robust data suggesting that T-DM1 is highly effective in this setting initially based on the EMILIA data [57], which compared T-DM1 with capecitabine and lapatinib. In the primary progression-free survival analysis of EMILIA [51], median DFS was 9.6 months in the trastuzumab emtansine group and 6.4 months in the capecitabine plus lapatinib group (hazard ratio 0·65 [95% CI 0.55–0.77]; *p* < 0.0001). T-DM1 also significantly increased overall survival (30.9 vs. 25.1 months; HR, 0.68; 95% CI, 0.55–0.85; *p* < 0.001). This benefit was observed across all subgroups, regardless of hormone receptor status and site of metastatic disease. Fewer grade 3 or worse adverse events were reported for trastuzumab emtansine versus capecitabine plus lapatinib (41% vs. 57%).

Therefore, only a small number of patients in EMILIA have received previous pertuzumab treatment. Even so, a limitation of the EMILIA study is that it does not provide evidence regarding the efficacy of trastuzumab emtansine after a patient has been treated with trastuzumab plus pertuzumab combination [57].

In the TH3RESA phase 3 trial, [58] has also shown improved DFS and OS with T-DM1 in patients with HER2-positive MBC who have been exposed to 2 anti-HER2 lines of therapy, including trastuzumab and lapatinib. T-DM1 improved the final overall survival compared with treatment of physician’s choice with a median overall survival pf 22.7 months vs. 15.8 months (HR 0.68 0.54–0.85; *p* = 0.0007).

Additional data for the use of trastuzumab emtansine in patients previously treated with HER2-targeted therapy plus chemotherapy will be obtained from real-world life studies. The Bahceci cohort [59] with 414 patients treated with T-DM1 in different lines, including anti HER2 therapy, shows an overall survival of 41 months, similar in first and second lines. However, in this cohort, there was a 30% of patients that are previously treated with lapatinib combinations and only 1% with pertuzumab combination. Battisti et al. [60] reported the Royal Marsden experience with T-DM1 in 128 patients with a 30% of previously treated with pertuzumab and shows a median disease-free survival and overall survival of 8 and 20 months respectively, and being very similar in the subgroup of patients who received prior treatment with pertuzumab.

The most important study to analyze T-DM1 after progression to previous treatments was the phase 3b KAMILLA study [61], with 2002 patients achieving a median disease-free survival and overall survival of 6.9 and 27.2 months respectively. Median DFS and OS decreased numerically with increasing prior lines of therapy. In patients with 0 to 1 prior lines of therapy, median DFS was 8.3 months, whereas in patients with 4 or more prior lines of therapy was 5.6 months. OS was 31.3 months in patients with 0 to 1 prior lines of therapy and 22.5 months in patients with 4 or more prior lines of therapy.

Table 3 summarizes the results of the most important trials of T-DM1 for previously treated metastatic breast cancer and the survival for the T-DM1 treated patients.

### 3.7. Treatment beyond Second Line

New agents have been developed in patients who had progressed on taxane, trastuzumab, pertuzumab, and T-DM1. Table 4 lists the most developed drugs that will be explained below.

#### 3.7.1. Lapatinib

Lapatinib was the first orally active small molecule used in anti-HER2 treatment, and reversibly inhibits HER2 and HER1 tyrosine kinases. Lapatinib plus capecitabine showed greater efficacy than capecitabine alone in patients with HER2-positive breast cancer who had previously been treated with at least one anthracycline, one taxane, and trastuzumab [62].

Another approach to use of lapatinib is to overcome resistance to endocrine therapy in HER2 positive patients by blocking the EGFR/HER2/ER pathway, combining lapatinib with endocrine therapy. In the EGF30008, patients were randomized to letrozole plus lapatinib and letrozole plus placebo, the median DFS was 8.2 vs. 3 months for the lapatinib arm. However this did not translate into an improvement in overall survival (33.3 vs. 32.3 months) [63].

The combination of lapatinib with trastuzumab was considered in the EGF104900 trial [64] in 291 heavily pretreated patients with an improvement in DFS (11.1 vs. 8.1 weeks) and overall survival (14 vs. 9.5 months). However, the value of this combination in patients previously treated with pertuzumab and T-DM1 is currently unknown so this combination has been displaced by the new drugs or new combinations.

#### 3.7.2. Neratinib

Neratinib is another small oral molecule that irreversibly inhibits HER1, HER2, and HER4. It has recently been approved as a third-line therapy based on the results of the phase III NALA trial [65]. In this trial, patients were randomized to receive capecitabine plus neratinib or capecitabine plus lapatinib. The neratinib arm showed a greater survival benefit with a median DFS of 8.8 months versus 6.6 months in the control arm. However, there were no statistically significant differences in OS between arms (HR, 0.88, 95% CI, 0.72–1.07, *p* = 0.2086). Notably, patients who received neratinib had a higher incidence of grade 3 diarrhea (24.4% vs. 12.5%), although with no impact on quality of life scores.

#### 3.7.3. Tucatinib

Tucatinib is a new TKI that is highly selective for the kinase domain of HER2 with minimal inhibition of EGFR. Tucatinib was evaluated in the HER2CLIMB study [66] in which patients who had progressed on a taxane, trastuzumab, pertuzumab, and T-DM1 were randomized to receive capecitabine with tucatinib and trastuzumab or capecitabine with trastuzumab. The triplet combination with tucatinib was associated with improved DFS and OS (7.8 vs. 5.6 months and 21.9 vs. 17.4 months). The most frequent adverse events were diarrhea, transaminitis, and hand-foot syndrome in the tucatinib arm, but a small number of patients had grade 3 or higher adverse events.

In this trial patients with untreated brain metastases were allowed, approximately half of the patients in HER2CLIMB had a history of brain metastases, and a survival benefit with the utilization of tucatinib was also seen in this population with a benefit in DFS of 7.6 months vs. 5.4 months. The risk of disease progression or death was 52% lower in the tucatinib-combination group than in the control arm group (hazard ratio, 0.48; 95% CI, 0.34 to 0.69; *p* < 0.001).

#### 3.7.4. Trastuzumab Deruxtecan

Trastuzumab deruxtecan is a new antibody-drug conjugate (ADC) composed of an antibody targeting HER2 that has a cleavable linker linked to a triple isomerase inhibitor. This ADC has a very high drug-to-antibody ratio (higher than seen with T-DM1) and has been associated with a 60% response rate in heavily pre-treated HER2-positive patients with a median of six prior lines of therapy. The DFS in this population was approximately 16 months, which is unprecedented in later-line HER2-positive therapy [67]. The long-term results were impressive, too, achieving a response duration of 14.8 months, median DFS of 16.4 months and the estimated OS of 93.9% at 6 months and 86.2% at 12 months. Adverse events were mild, the most common were gastrointestinal and hematological toxicity, however there is a high incidence of interstitial lung disease (13.6% with 0.5% grade 4) that should be taken into account

Currently, FDA approval is based on a single-arm study of trastuzumab deruxtecan, in the third line and beyond. At present, there are a wide series of studies with trastuzumab deruxtecan in the different lines of treatment within the DESTINY program. We’re awaiting results from a trial that had compared trastuzumab deruxtecan with T-DM1 in the second-line setting, which will help us better understand if we can utilize this agent after progression to trastuzumab and pertuzumab combinations.

#### 3.7.5. Margetuzimab

Margetuximab is a new monoclonal anti-HER2 antibody with an Fc fraction derived from trastuzumab whereby it binds to the same HER2 receptor epitope. Margetuximab has an immunoglobulin 1 Fc region that is genetically modified for a higher affinity of the stimulating receptor FcgR IIIA and a lower affinity for the inhibitory receptor FcgR IIB on NK cells, thus increasing antibody-dependent cellular cytotoxicity [68].

Margetuximab was evaluated in the phase III SOPHIA trial [69], with 536 patients with HER2-positive metastatic breast cancer who had progressed to at least two lines of anti-HER2 therapy, including pertuzumab, margetuximab, or trastuzumab, both combined with chemotherapy. Margetuximab-treated patients had a survival benefit with a median DFS of 5.8 months versus 4.9 months in the control arm with physical choice treatment. No significant improvement in overall survival was achieved. However, in the subgroup analysis, it was observed that patients with the CD16A genotype that contain a 158F allele, presented a greater efficacy also in overall survival. The safety analysis showed a comparable safety margin in both arms.

#### 3.7.6. Pyrotinib

Pyrotinib is another new irreversible small molecule pan-HER TKI receptor. It has been evaluated in the PHOEBE phase III clinical trial [70] with 267 patients with HER2-positive advanced breast cancer previously treated with trastuzumab and taxanes and/or anthracyclines to receive pyrotinib or lapatinib with capecitabine. There is a survival benefit with a median DFS of 12.5 months for pyrotinib plus capecitabine versus 6.8 months for lapatinib plus capecitabine (HR: 0.39, *p* < 0.001). The most common grade 3 adverse events were diarrhea, which was more common in the pirotinib group (30.6% vs. 8.3%), and hand-foot syndrome, although there were no differences between the two arms (16.4%). versus 15.2%).

There was a different population in this trial than in the other new drugs reported trials because 39% and 45% of patients were first- and second-line treated, respectively, which explains the better survival found. Overall survival data were not mature, however, there was a strong trend towards an improved OS in the pyrotinib arm (HR 0.46; 95% CI 0.22–0.99). There is also an extensive pyrotinib development program in China. With several phase 2 and 3 trials in different disease stages (NCT04033172, NCT0 3933982, NCT03910712, NCT03876587, NCT04254263, NCT04255056, NCT04126525, NCT03735966, NCT04407988, NCT03863223, NCT03588091, NCT04290793).

#### 3.7.7. Trastuzumab Duocarmazime

Another targeting ADC that comprises an HER2 antibody similar to trastuzumab conjugated with alkylator agent duocarmycin is Trastuzumab duocarmazine (SYD985) In a phase I trial [71] with HER2-positive advanced disease, the ORR was 33%. The most common treatment-related adverse events were fatigue (33%), conjunctivitis (31%) and dry eyes (31%). Almost two thirds of patients (71%) had at least one ocular adverse event, with grade 3 events reported in 7% of patients. Four patients in the whole cohort developed pneumonitis, one of them even a grade 4 pneumonitis (1%). The TULIP phase III trial of SYD985 versus trastuzumab and chemotherapy in metastatic HER2-positive breast cancer is currently ongoing.

#### 3.7.8. Palbociclib and Abemaciclib

The association between HER2 signaling and regulation of cyclin D1-CDK complexes has been established in preclinical studies [72]. In the phase II clinical trial MonarchHER [73], the combination of endocrine therapy plus CDK 4/6 inhibitor and anti-HER2 treatment was studied. This trial included 237 patients with HER2 positive and HR positive advanced breast cancer who had previously been treated with at least two antiHER2 targeted therapies of abemaciclib, trastuzumab and fulvestrant, abemaciclib with trastuzumab, or chemotherapy plus trastuzumab. Efficacy results were superior for the triple combination with a median DFS of 8.3 months versus 5.7 for the chemotherapy plus trastuzumab combination.

Another study conducted with CDK4/6 inhibitors was the PATRICIA phase II trial [74] with 71 patients who had received 2–4 previous lines of anti-HER2-based regimens to receive palbociclib plus trastuzumab with or without letrozole (if Positive HR). The benefit of palbociclib and trastuzumab was restricted for HR-positive patients. But the most relevant aspect of this study is that it was found that the luminal intrinsic subtype determined by PAM50 presented a greater benefit with a DFS of 10.6 months compared to 4.2 months. Based on these results, the PATRICIA-II phase II trial (NCT02448420) was started, comparing palbociclib, trastuzumab, and endocrine therapy versus chemotherapy and trastuzumab in patients with luminal disease by the PAM50 genomic test. In Table 5 are resumed the main trials in this setting.

**Figure 4:**
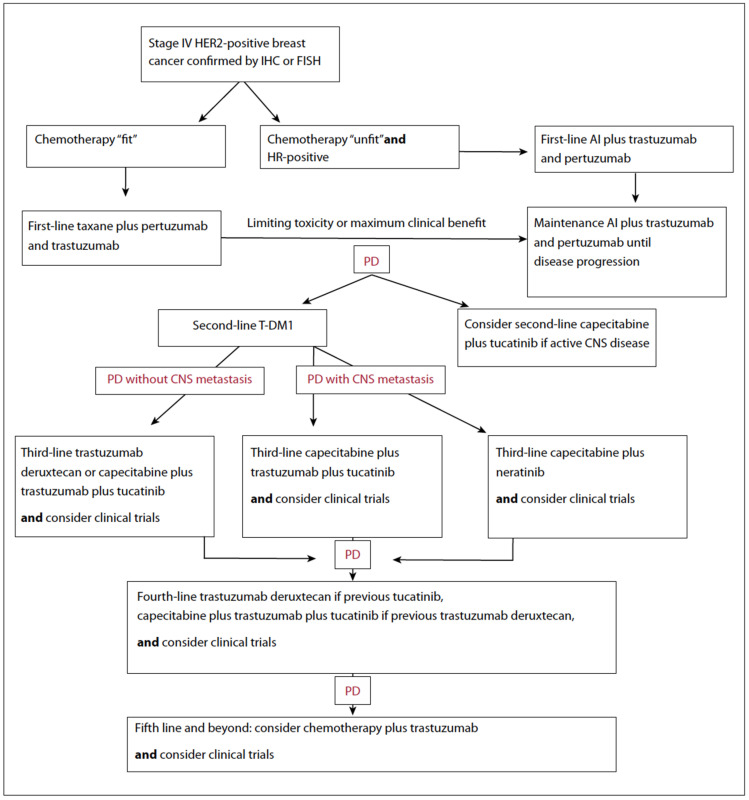
Proposed treatment algorithm for HER2+ metastatic breast cancer.

A final comprehensive approach to the treatment of stage IV HER2-positive breast cancer is proposed in Figure 4. Note that more options will be based on the different regulations of the competent health authorities.

## 4. Conclusions

Prognosis of the HER2 positive breast cancer subtype has significantly improved, on the one hand due to a greater understanding of its intrinsic molecular biology and, on the other, due to the discovery of new targeted therapies. Therefore, it is very important to confirm the molecular diagnosis in order to offer these specific treatments. There are computer systems that can help us and greatly improve our objectivity.

In early disease, the neoadjuvant approach obtains great significance as it indicates how to continue with the different treatment options, achieving high rates of pathological response and survival. In metastatic disease, long survival times have been achieved and the new, powerful drugs developed that allow us to chain these treatments in case of progression or incorporate them to earlier stages to achieve even greater benefit.

## Figures and Tables

**Table 1:** With all this information we propose a treatment algorithm to sum up all recommendations.

SITUATION	NEOADJUVANT	pCR	ADJUVANT
T < 2 cm, N0	12 weeks paclitaxel + trastuzumab	pCR YES	Continue trastuzumab to complete 1 year +/− endocrine therapy if HR+
		pCR NO	HR+: consider 1 year of trastuzumab + 1 year of Neratinib + endocrine therapy
			HR+/HR-: consider adjuvant TDM1
T > 2 cm and or N+	4 cycles of antrhacyclines + ciclophosphamide + 12 weeks of paclitaxel + trastuzumab + pertuzumab Or carboplatin + paclitaxel + trastuzumab + pertuzumab x 6 cycles	pCR YES	Continue trastuzumab to complete 1 year +/− endocrine therapy if HR+
		pCR NO	HR+: consider 1 year of trastuzumab + 1 year of Neratinib + endocrine therapy
			HR+/HR-: consider adjuvant TDM1
T < 2 cm, N0	Not Receiving Neoadjuvant Treatment		12 weeks of paclitaxel + trastuzumab and continue trastuzumab to complete 1 year +/− endocrine therapy if HR+
T > 2 cm, N0	Not Receiving Neoadjuvant Treatment		4 cycles of antrhacyclines + ciclophosphamide + 12 weeks of paclitaxel + trastuzumab and continue trastuzumab to complete 1 year +/− endocrine therapy if HR+Consider + 1year of Neratinib if HR + and high risk tumor
T > 2 cm, N+	Not Receiving Neoadjuvant Treatment		4 cycles of antrhacyclines + ciclophosphamide + 12 weeks of paclitaxel + trastuzumab + pertuzumab and continue trastuzumab + pertuzumab to complete 1 year +/− endocrine therapy if HR+Consider + 1 year of Neratinib if HR + and high risk tumor

pCR: pathologic complete response.

**Table 2:** New approaches in first line.

Clinical Trial	Phase	Treatment Arm Study	Conditions	Enrollment
**NCT04246502**	Phase II	Capecitabine plus pyrotinib	First Line	200
**NCT03199885**	Phase III	Atezolizumab	First Line	600
**NCT04784715**	Phase III	Trastuzumab deruxtecan	First Line	1134
**NCT03910712**	Phase II	Pyrotinib plus aromatase inhibitor	HR positive	250
**NCT04088110**	Phase II	Pyrotinib plus aromatase inhibitor	HR positive	77
**NCT04760431**	Phase II	Pyrotinib or tucatinib	Brain metastases	120
**NCT04263298**	Phase III	Fulvestrant	Maintenance	368
**NCT03304080**	Phase I/II	Palbociclib	HR positive	36
**NCT 02947685**	Phase III	Palbociclib	Maintenance	496
**NCT04253561**	Phase Ib	Ipatasertib	Maintenance	25

**Table 3:** Main trials of TDM-1.

Trial	Type	Size	Previous Treated	DFS	OS
**EMILIA**	Phase III	991	Trastuzumab and taxane	9.6	30.9
**TH3RESA**	Phase III	602	Trastuzumab and lapartinib	6.2	22.7
**Baheci**	RWD	414	37% in second line	12	41
**Battisti**	RWD	128	30% with pertuzumab	8	20
**KAMILLA**	Phase IIIb	2020	22% in second line	6.9	27.2

**Table 4:** New drugs after second line treatment.

Drugs	Family
**Lapatinib**	TKI
**Neratinib**	TKI
**Tucatinib**	TKI
**Trastuzumab deruxtecan**	ADC
**Margetuximab**	Monlclonal Antibody
**Pyrotinib**	TKI
**Trastuzumab duocarmazine**	ADC
**Palbociclib and abemaciclib**	CDH 4/6 inhibitors

**Table 5:** Summarized results of the most important trials with new drugs.

Trial	Drugs	Control Arm	DFS	OS
**EGF30008**	Lapatinib	Letrozol	8.2 m	32 m
**EGF104900**	Lapatinib + trastuzumab	lapatinib	12 w	51 w
**NALA**	Neratinib + Capecitabine	lapatinib + capecitabine	8.8 m	24 m
**HER2CLIMB**	Tucatinib + Capecitabine	trastuzumab + capecitabine	7.8 m	21.9 m
**DESTINY**	Trastuzumab deruxtecan	none, Phase II	16 m	NA
**SOPHIA**	Margetuximab + Chemotherapy	Trastuzumab + chemotherapy	5.8 m	21.6 m
**PHOEBE**	Pyrotinib + Capecitabine	lapatinib + capecitabine	12.5 m	NA
**TULIP**	Trastuzumab duocarmazine	none, Phase II	7.6 m	NA
**MonarHER**	Abemaciclib + ET + Trastuzumab	Trastuzumab + chemotherapy	8.3 m	NA
**PATRICIA**	Palbociclib + ET + Trastuzumab	none, Phase II	10.6 m	NA
